# NULISA analysis in genetic frontotemporal dementia: a study on the GENetic FTD Initiative

**DOI:** 10.1002/alz70861_108554

**Published:** 2025-12-23

**Authors:** Aitana Sogorb‐Esteve, Irene Gorostiaga‐Belio, Katie Tucker, Owen Swann, GENFI consortium, Amanda J Heslegrave, Maryam Shoai, Henrik Zetterberg, Jonathan D. Rohrer

**Affiliations:** ^1^ Dementia Research Centre at University College London, London UK; ^2^ UK Dementia Research Institute at University College London, London UK; ^3^ Dementia Research Centre at UCL, London UK; ^4^ UK Dementia Research Institute at UCL, London UK; ^5^ GENFI Consortium, GENFI UK; ^6^ Clinical Neurochemistry Laboratory, Sahlgrenska University Hospital, Mölndal, Västra Götalands län Sweden; ^7^ Dementia Research Centre, Department of Neurodegenerative Disease, UCL Queen Square Institute of Neurology, University College London, London, London UK

## Abstract

**Background:**

Given the clinical and pathological heterogeneity of frontotemporal dementia (FTD), there remains a critical need for tools that support the development and implementation of sensitive and specific fluid biomarkers. Emerging technologies, such as NULISA (Nucleic Acid Linked Immunosorbent Assay), offer the potential for improved detection capabilities. In this study, we evaluated the NULISA CNS panel in plasma samples from participants in the Genetic FTD Initiative (GENFI) cohort to explore its utility in the context of FTD.

**Method:**

We used the CNS NULISA panel containing 124 targets in plasma samples from the GENFI cohort. We measured a total of 743 participants including 238 *C9orf72* expansion carriers (151 presymptomatic and 87 symptomatic), 166 *GRN* mutation carriers (113 presymptomatic and 53 symptomatic), 100 *MAPT* mutation carriers (63 presymptomatic and 37 symptomatic), and 239 non‐carriers as controls. Linear mixed models were used to identify protein changes between the groups.

**Result:**

Preliminary analysis reveals several significant differences between clinical groups and controls. As expected NEFL and GFAP show strong association with symptomatic individuals versus controls (adjusted *p* <0.001 for both). Notably, NPTXR and SNAP25 are significantly reduced in symptomatic participants compared to controls (FDR adjusted *p* <0.001 and *p* =0.003, respectively) and appear to have a genotype specific effect. NPTXR was reduced in *GRN*, *C9orf72* and *MAPT* positive symptomatic individuals versus controls, whilst SNAP25 reduction appears more specific to GRN mutation carriers. Further analyses are underway to elucidate group differences and changes at presymptomatic stages as well as within pathology specific groups.

**Conclusion:**

Our preliminary results show promising potential for the NULISA technology in the context of frontotemporal dementia and further analysis underway will provide useful information for the biomarker field.

